# Lrig1 Expression Defines a Distinct Multipotent Stem Cell Population in Mammalian Epidermis

**DOI:** 10.1016/j.stem.2009.04.014

**Published:** 2009-05-08

**Authors:** Kim B. Jensen, Charlotte A. Collins, Elisabete Nascimento, David W. Tan, Michaela Frye, Satoshi Itami, Fiona M. Watt

**Affiliations:** 1 Laboratory for Epidermal Stem Cell Biology, Wellcome Trust Centre for Stem Cell Research, University of Cambridge, Cambridge CB2 1QR, UK; 2 Laboratory for Epithelial Stem Cell Homeostasis and Cancer, Wellcome Trust Centre for Stem Cell Research, University of Cambridge, Cambridge CB2 1QR, UK; 3 Department of Regenerative Dermatology, Graduate School of Medicine, Osaka University, 2-2, Yamadaoka, Suita-shi, Osaka 565-0871, Japan; 4 Epithelial Cell Biology Laboratory, Cancer Research UK Cambridge Research Institute, Li Ka Shing Centre, Cambridge CB2 0RE, UK

**Keywords:** STEMCELL

## Abstract

Lrig1 is a marker of human interfollicular epidermal stem cells and helps maintain stem cell quiescence. We show that, in mouse epidermis, Lrig1 defines the hair follicle junctional zone adjacent to the sebaceous glands and infundibulum. *Lrig1* is a Myc target gene; loss of *Lrig1* increases the proliferative capacity of stem cells in culture and results in epidermal hyperproliferation in vivo. Lrig1-expressing cells can give rise to all of the adult epidermal lineages in skin reconstitution assays. However, during homeostasis and on retinoic acid stimulation, they are bipotent, contributing to the sebaceous gland and interfollicular epidermis. β-catenin activation increases the size of the junctional zone compartment, and loss of *Lrig1* causes a selective increase in β-catenin-induced ectopic hair follicle formation in the interfollicular epidermis. Our results suggest that *Lrig1*-positive cells constitute a previously unidentified reservoir of adult mouse interfollicular epidermal stem cells.

## Introduction

Homeostasis in adult tissues requires balanced proliferation and differentiation. In many cases, this depends on resident stem cells that self-renew and produce the appropriate differentiated lineages of the tissue (Morrison and Spradling, 2008). Stem cells reside in unique microenvironments, niches, that are required to maintain the cells' unique properties (Fuchs et al., 2004; Watt and Hogan, 2000).

Mammalian epidermis comprises the interfollicular epidermis (IFE) with associated hair follicles (HF), sebaceous glands (SG), and sweat glands. There are believed to be distinct populations of stem cells in different locations. The lineages that they feed are normally constrained by signals from their local environment, but they can give rise to all epidermal lineages in response to appropriate stimuli (Chuong, 2007; Jones et al., 2007; Owens and Watt, 2003).

In human IFE, clusters of stem cells have been identified on the basis of long-term self-renewal ability in culture and quiescence in vivo; these cells express a range of markers, including high levels of β1 integrins (Gambardella and Barrandon, 2003; Watt, 2002). In contrast, in mouse epidermis, the best characterized stem cell population is in a region of the HF known as the bulge; these cells express keratin 15, CD34, and LGR5 and include a subpopulation of infrequently dividing, DNA label-retaining cells (LRC) (Cotsarelis et al., 1990; Jaks et al., 2008; Liu et al., 2003; Trempus et al., 2003). Recent lineage tracing has established that mouse IFE can be temporarily reconstituted from bulge stem cells (Ito et al., 2005; Levy et al., 2007) but is maintained by actively cycling cells that divide or differentiate according to stochastic principles (Clayton et al., 2007). Thus, the mouse IFE stem cell compartment has proved elusive.

In a screen for human epidermal stem cell markers, we identified Leucine-rich repeats and immunoglobulin-like domain protein 1 (LRIG1) (Jensen and Watt, 2006). LRIG1 is a transmembrane protein that interacts with and decreases signaling by ErbB growth factor receptors (Gur et al., 2004; Jensen and Watt, 2006; Laederich et al., 2004).

Expression of Lrig1 is required for epidermal homeostasis, as genetic deletion of *Lrig1* leads to epidermal hyperplasia in mouse skin (Suzuki et al., 2002). Knockdown of *Lrig1* in cultured human keratinocytes causes increased proliferation associated with stem cell expansion (Jensen and Watt, 2006). In cultured keratinocytes, LRIG1 negatively regulates EGF-induced ERK MAPK signaling and decreases *cMyc* transcription, RNA, and protein levels (Jensen and Watt, 2006). Because epidermal overexpression of Myc can stimulate both proliferation and terminal differentiation (Arnold and Watt, 2001; Waikel et al., 2001), it has been proposed that Myc-induced differentiation acts as a fail-safe device to prevent uncontrolled proliferation of stem cells (Jensen and Watt, 2006).

We have now examined Lrig1 expression and function in mouse epidermis. We report that Lrig1 expression defines a previously uncharacterized multipotent stem cell population, which normally contributes to the IFE and SG lineages.

## Results

### Lrig1 Expression Specifies a Unique Population of Epidermal Cells during Development

**Figure 1:**
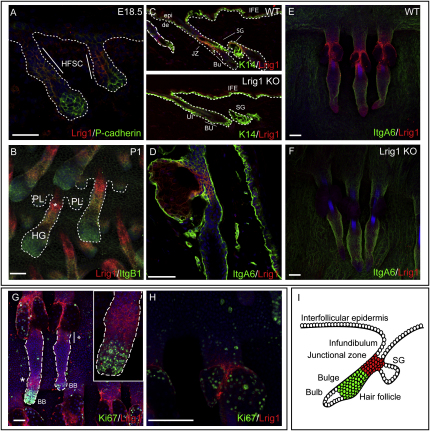
Lrig1 Expression Defines a Distinct Population of Epidermal Cells in the Junctional Zone (A–H) Immunofluorescence labeling of sections of embryonic (A) and adult back (C) and tail (D) skin and whole mounts of embryonic (B) and adult (E–H) tail epidermis. Adult skin was in telogen (C–F and H) or early anagen (G). Skin was from wild-type (A, B, upper panel of C, D, E, G, and H) or *Lrig1*-null mice (lower panel of C, F). Insert in (G) shows the bulb at a higher magnification. Color coding indicates antibody labeling. Dashed lines represent the boundary between dermis and epidermis (A and C) or demarcate hair follicles (B and G). epi, epidermis; de, dermis; IFE, interfollicular epidermis; SG, sebaceous gland; JZ, junctional zone; BU, bulge; BB, bulb; HFSC, developing hair follicle stem cell compartment; PL, hair follicle placode; HG, hair germ. (A) is shown at a higher magnification in Figures S1F–S1H. Scale bars, 25 μm (A and D) and 100 μm (B and E–H). (I) Diagram illustrating the different regions of adult epidermis. Red, junctional zone; green, bulge.

At E14.5, prior to HF placode formation, Lrig1 was expressed at low levels in dorsal epidermis and at higher levels by a subpopulation of dermal cells (Figure S1A available online). At E17.5 and E18.5, Lrig1 expression was upregulated in the P-cadherin dim population of multipotent stem cells in the developing HF (Nowak et al., 2008) and was absent from the P-cadherin bright cells at the base (Figures 1A, S1B, and S1F–S1H). Thus, during development, the presumptive bulge stem cell population expressed Lrig1. This expression pattern persisted at P1, except that from then onward, dermal expression was reduced (Figure S1C). Q-PCR of RNA isolated from back skin samples (Figure S1D) revealed that *Lrig1* levels peaked at P1.

In whole mounts of tail epidermis (Braun et al., 2003), the distinct location of the Lrig1-positive population was visualized readily at P1 and P5. β1 integrin is highly expressed by the developing hair follicle stem cell population, expression extending distally from the bulge to the HF bulb (Figures 1B and S1E). Lrig1-expressing cells in the upper part of the follicle expressed low levels of β1 integrins. By P5, tail epidermal sebaceous glands start to develop; they arose from the Lrig1-positive region of the HF and were themselves Lrig1 positive (Figure S1E, asterisk).

### Lrig1 Expression Defines a Distinct Population of Cells in Adult Epidermis

In adult telogen back and tail epidermis (resting phase of the hair growth cycle), Lrig1 expression defined a distinct population of cells at the junction between the infundibulum and the SG (Figures 1C–1F), but Lrig1 was no longer detectable in the SG (Figure 1C). Lrig1 is the first reported marker of the junctional zone between the HF bulge, SG, and infundibulum (Figure 1I).

Whereas Lrig1-positive cells in the junctional zone were present throughout the hair growth cycle, two additional sites of Lrig1 expression were found exclusively in anagen (growing) follicles. Lrig1-positive cells were detected in the outer-root sheath below the level of the SGs (Figure 1G, bar and asterisk), extending toward the bulge. They were also found in a ring of cells above the bulb (Figure 1G, asterisk and insert).

**Figure 2:**
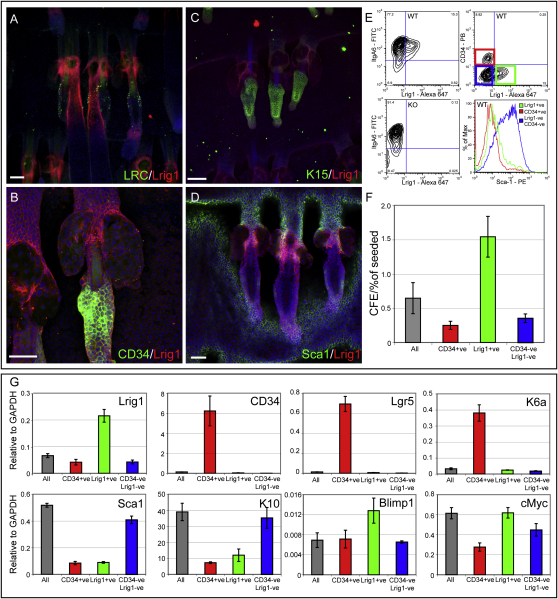
Characterization of Lrig1-Expressing Cells in Adult Epidermis (A–D) Immunofluorescence labeling of tail epidermal whole mounts from wild-type mice. Skin was in anagen (A) or telogen (B–D). Color coding indicates antibody labeling. Scale bars, 100 μm. (E) Flow cytometry of telogen epidermis from wild-type (WT) and *Lrig1*-null (KO) epidermis disaggregated with thermolysin and labeled with antibodies to α6 integrin, Lrig1, CD34, and Sca1. Colored gates in top-right panel correspond to populations in lower-right panel and in (F) and (G). (F) Colony-forming efficiency of wild-type primary keratinocytes isolated as in (E). (G) Q-PCR of RNA from 10^5^ wild-type epidermal cells isolated as in (E). (F and G) Data are means ± SEM (n = 4).

Lrig1-expressing cells in adult mouse epidermis were largely quiescent. In anagen, the zone of Lrig1 expression above the bulb separated the highly proliferative bulb cells from the rest of the outer-root sheath (Figure 1G). In the junctional zone of Lrig1-positive cells, there were fewer Ki67-positive cells than in the adjacent SGs (Figure 1H). In late anagen, the Lrig1-positive cells below the level of the SGs included DNA label-retaining cells, another indicator of quiescence (Braun et al., 2003; Figure 2A).

We compared Lrig1 expression in telogen epidermis with expression of markers previously reported to define bulge stem cells (CD34, Keratin 15, high levels of α6 integrin) (Lyle et al., 1998; Tani et al., 2000; Trempus et al., 2003). Lrig1-positive cells expressed high levels of α6 integrin (Figures 1D–1F) but were negative for CD34 (Figure 2B). They lacked Keratin 15 (Figure 2C) and expressed low levels of Sca1, which is highly expressed in the infundibulum and IFE (Jensen et al., 2008; Figure 2D).

### Isolation of Lrig1-Expressing Cells

To test the ability of Lrig1-positive cells to self-renew and contribute to different epidermal lineages, we used flow cytometry to isolate cells expressing Lrig1. The Lrig1 extracellular domain is sensitive to the trypsinization conditions normally used to disaggregate epidermal cells; however, the Lrig1 epitope was retained when cells were disaggregated with thermolysin (Figure 2E and data not shown). When disaggregated epidermal cells were double-labeled with antibodies to α6 integrin and Lrig1, the Lrig1-positive population had high α6 levels (Figure 2E), consistent with the immunolocalization data (Figures 1C–1E). The specificity of Lrig1 labeling was demonstrated by frozen section and whole-mount immunostaining and flow cytometry of cells from *Lrig1*-null mice (Figures 1C, 1F, and 2E). As expected (Figures 2B and 2D), Lrig1-positive cells lacked CD34 and had low levels of Sca1 (Figure 2E).

To examine clonal growth capacity, undifferentiated, α6-positive basal cells (low forward and side scatter; all) were sorted into different fractions: α6, CD34 double-positive (CD34+); α6, Lrig1 double-positive (Lrig1+); and α6-positive, CD34, Lrig1 double-negative (CD34-Lrig1−) cells (Figure 2F). Only the Lrig1-positive population was enriched for clonal growth ability relative to unselected α6-positive cells (Figure 2F). Thus, Lrig1 expression defines a distinct population of highly clonogenic epidermal cells.

We next analyzed RNA from flow-sorted cells (Figure 2G). As predicted, Lrig1-expressing cells did not express the bulge markers *CD34* and *Lgr5* (Jaks et al., 2008; Trempus et al., 2003). They also lacked hair keratin 6a, a marker of cells in the bulge and outer-root sheath (Gu and Coulombe, 2007). Lrig1-positive cells expressed low levels of *Sca1* and the IFE-terminal differentiation marker keratin 10. Lrig1-positive cells were enriched for expression of the putative SG stem cell marker *Blimp1* (Horsley et al., 2006; Lo Celso et al., 2008) and had higher *cMyc* expression than did CD34+ cells. Thus, Lrig1-expressing cells are transcriptionally distinct from bulge stem cells and committed HF and IFE cells.

**Figure 3:**
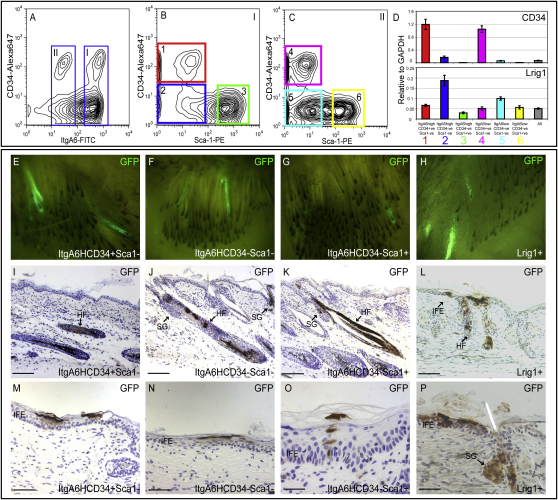
Epidermal Reconstitution by Lrig1-Expressing Cells (A–C) Keratinocytes isolated from the back skin of wild-type adult telogen (7-week-old) mice were sorted into α6 integrin-high (I) and -low (II) populations (A). Populations I (B) and II (C) were gated into three further populations on the basis of Sca1 and CD34 expression, yielding a total of six discrete populations of keratinocytes (1–3 in [B]; 4–6 in [C]). (D) RNA from each of the six populations or total live cells (low forward/side scatter; all) were analyzed using Q-PCR for the genes indicated (mean ± SEM, n = 5). (E–P) Epidermal cells from telogen back skin of GFP-expressing mice were fractionated based on Sca1, α6 integrin, and CD34 expression as in (A)–(C) or Lrig1 (as in Figure 2E) for epidermal reconstitution experiments by mixing GFP-positive and GFP-negative epidermal cells. Four cell populations were compared: α6 high (ItgA6H), CD34 positive, Sca1 negative (population 1; enriched for bulge cells; [E], [I], and [M]); α6 high, CD34 negative, Sca1 negative (population 2; enriched for Lrig1-positive cells; [F], [J], and [N]); α6 high, CD34 negative, SCA1 positive (population 3; [G], [K], and [O]); or Lrig1 positive ([H], [L], [P]). (E–H) Dermal view of grafts; (I-P) Sections of grafts labeled with GFP antibody (brown) and hematoxylin counterstain (blue). Interfollicular epidermis (IFE), hair follicles (HF), and sebaceous glands (SG) are indicated. Scale bars, 100 μm (I–L) and 25 μm (M–P).

To allow isolation of junctional zone cells from trypsinized and *Lrig1*-null epidermis, we developed a second sorting strategy. Undifferentiated (low forward and side scatter) epidermal keratinocytes were sorted into six distinct populations based on CD34, α6 integrin, and Sca1 expression (Jensen et al., 2008; Figures 3A–3C). Epidermal cells were divided into α6 integrin-high (I in Figure 3A) or -low (II in Figure 3A) populations (Blanpain et al., 2004; Jensen et al., 2008; Silva-Vargas et al., 2005). These two populations were each fractionated into three further groups—CD34 positive, Sca1 negative (1 in Figure 3B; 4 in Figure 3C); CD34 negative, Sca1 negative (2 in Figure 3B; 5 in Figure 3C); or CD34 negative, Sca1 positive (3 in Figure 3B; 6 in Figure 3C)—and RNA was isolated from all six populations. The color coding of the different populations in Figures 3B and 3C matches the colors in Figure 3D. Cells in population 2 were, as expected, enriched for *Lrig1* expression (Figures 3A–3D and Figures S2A–S2F) and had the same RNA expression profile as cells sorted with Lrig1 antibodies (Figures S2D–S2F; cf. Figure 2G). We conclude that sorting CD34-negative, Sca1-negative, α6 integrin-high cells represents an alternative strategy for enriching *Lrig1*-positive cells.

### Lrig1-Expressing Cells Can Contribute to All Epidermal Lineages

To examine the capacity of Lrig1-positive cells to generate different epidermal lineages, we isolated cells on the basis of CD34, Sca1, and α6 integrin expression from mice expressing GFP under the control of the *chicken β-actin promoter*. The 10^5^ sorted GFP-positive cells were combined with 3 × 10^6^ unlabeled, unfractionated epidermal cells from a wild-type (GFP-negative) mouse. The low ratio of GFP-positive to GFP-negative cells allowed subsequent clonal analysis. The disaggregated epidermal cells were combined with P2 dermal cells, injected into a silicone chamber on the back of a nude mouse, and allowed to reconstitute skin for 4–5 weeks. We compared bulge cells (CD34 positive, Sca1 negative, α6 high) (population 1 in Figure 3B) with Lrig1-enriched cells (CD34 negative, Sca1 negative, α6 high) (population 2 in Figure 3B) and CD34-negative, Sca1-positive, α6-high cells (population 3 in Figure 3B), which have previously been reported to be lineage-committed IFE cells (Jensen et al., 2008). We also isolated Lrig1, GFP double-positive cells from thermolysin-treated epidermis using Lrig1 antibodies and combined them with unfractionated, trypsin-disaggregated cells.

At 4–5 weeks postgrafting, grafts were analyzed for the contribution of GFP-positive cells to SGs, HFs, and IFE. When viewed macroscopically from the dermal side, the contribution of all four populations of GFP-positive cells to HFs was evident (Figures 3E–3H). The extent of contribution of each population was similar: 6% ± 4% of HFs contained GFP-positive, Lrig1-enriched cells, compared to 1% ± 1% for CD34-positive cells and 3% ± 1% for Sca1-positive cells (n = 3 ± SEM). The number of reconstituted follicles was lower following thermolysin treatment, reflecting decreased cell viability (Figure 3H and data not shown).

By staining sections with GFP antibodies, we established that all four populations founded IFE clones of varying size and contributed to HFs and SGs (Figures 3I–3P). All HFs and SGs that contained GFP-positive cells also had a contribution of GFP-negative cells (Figures 3I–3L and 3P). None of the sorted populations were restricted in terms of contribution to the different HF lineages (Figures 3I–3L and 3P).

We conclude that populations of bulge stem cells, Lrig1-positive cells, and the Sca1-positive cells previously reported to be committed IFE cells (Figure 2D) all contain multipotent cells that contribute to all lineages in reconstituted epidermis.

### Lrig1 Expression Controls Proliferation in the Interfollicular Epidermis

**Figure 4:**
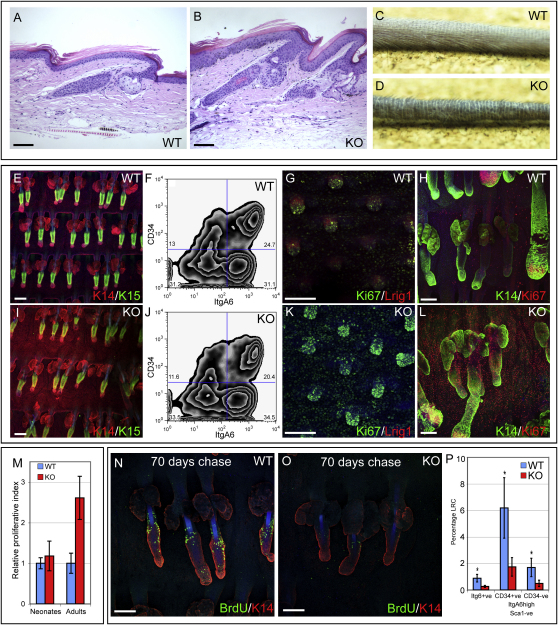
Effects of *Lrig1* Loss In Vivo (A and B) Hematoxylin and eosin-stained sections of adult tail skin. (C and D) Macroscopic view of adult tails. (E, I, G, H, K, L, N, and O) Tail whole mounts of P1 (G and K) and adult telogen (E, I, N, and O) and anagen (H and L) epidermis. WT, wild-type; KO, *Lrig1* null. Color coding indicates antibody labeling. (F and J) Flow cytometry of (F) WT and (J) KO Sca1-low cells with antibodies to CD34 and α6 integrin. The percentage of cells in each quadrant is indicated. (M) Quantitation of Ki67-positive cells in KO IFE expressed relative to WT controls. Data shows mean ± SEM; average of three fields per whole mount and five mice per sample. (N–P) BrdU label-retaining cells were examined following a 70 day chase period. (P) Cells were subjected to flow cytometry with antibodies to Sca1, CD34, and α6 integrin. The percentage of cells in each group that were BrdU labeled is shown. Error bars represent SD (WT n = 8; KO n = 6). WT, wild-type; KO, *Lrig1* null. Scale bars, 100 μm (A, B, G, H, K, M, and O) and 200 μm (E and I).

We next generated *Lrig1*-null mice by breeding heterozygous animals and compared null, heterozygous, and wild-type littermates. We saw no differences between wild-type and heterozygous animals. The tail epidermis of *Lrig1*-null mice was thicker than that of wild-type (Figures 4A and 4B), and HFs protruded at a greater angle (Figures 4C and 4D). There was no SG hyperplasia, and the hair growth cycle was not grossly perturbed (Figures 4A and 4B and data not shown). Expression of Keratin 10 and Keratin 15 was normal (Figures 4E and 4I and data not shown). As reported previously, Keratin 6 was expressed in *Lrig1*-null IFE, consistent with epidermal hyperproliferation (Suzuki et al., 2002; data not shown). Flow cytometry for Sca1, CD34, and α6 integrin (Figures 4F and 4J and data not shown) demonstrated that loss of *Lrig1* did not affect the size of any of the six cell populations defined in Figures 3A–3C. Thus, loss of *Lrig1* did not change the size of the bulge stem cell compartment or the population of cells that normally express Lrig1.

The most striking phenotype was an increase in IFE proliferation. In embryonic and P1 epidermis, the effect was evident in some areas (Figures 4G and 4K), but the mean data from replicate samples was not statistically significant (Figure 4M). However, in adult skin, there was a marked increase both in Ki67-positive cells and in BrdU-labeled S phase cells (Figures 4H, 4L, 4M, and data not shown).

Consistent with increased epidermal proliferation, the number of DNA label-retaining cells in adult *Lrig1*-null epidermis was substantially reduced (Figures 4N and 4O). This was quantitated by flow cytometric analysis of label-retaining cells in the bulge (CD34 positive, Sca1 negative, α6 high), the cells that would normally express Lrig1 (CD34 negative, Sca1 negative, α6 high), and total undifferentiated epidermal cells (α6 positive) (Figure 4P). The number of label-retaining cells was decreased in all three populations.

These results demonstrate that Lrig1 is required for stem cell quiescence in adult mouse epidermis, as observed in cultured human epidermis (Jensen and Watt, 2006).

### Lrig1 and cMyc Form an Autoregulatory Feedback Loop

**Figure 5:**
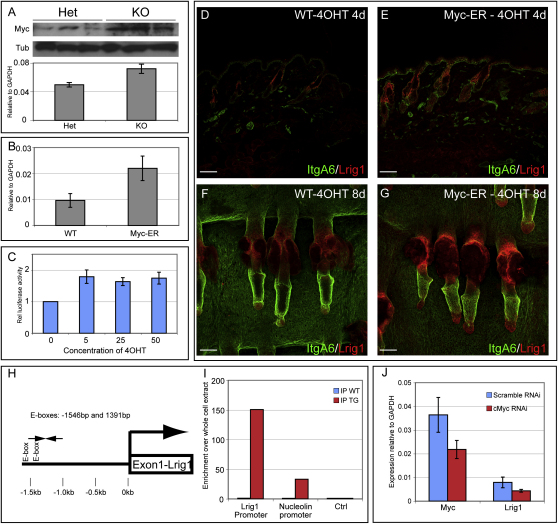
Regulation of Lrig1 Expression by Myc (A) (Top) Immunoblot of protein lysates from tail skin of adult littermates lacking (KO) or heterozygous (Het) for *Lrig1*. Each lane contains protein from a separate mouse. Blot was probed for Myc or, as a loading control, β-tubulin (Tub). (Bottom) Q-PCR for *cMyc* from adult littermates lacking (KO) or heterozygous (Het) for *Lrig1*. Expression levels are relative to *Gapdh*, and error bars represent SD (n = 3). (B) Q-PCR for *Lrig1* mRNA in back skin of WT and *K14MycER*-transgenic mice treated with 4OHT for 8 days. Expression levels are relative to *Gapdh*, and error bars represent SD (n = 3). (C) Luciferase assay using 1.6 kb of the *Lrig1* promoter in murine keratinocytes from WT or *K14MycER* mice, represented as induction in *K14MycER* cells relative to WT cells. Cells were treated with the 4OHT concentrations indicated (nM). Error bars represent SD (n = 4). (D–G) Back skin sections (D and E) and tail epidermal whole mounts (F and G) of wild-type (WT) and *K14MycER* mice treated with 4OHT for the number of days shown. Color coding indicates antibody labeling. Scale bars, 100 μm. (H) Location of two E boxes in the *Lrig1* promoter. (I) Quantitative ChIP from keratinocytes isolated from back skin of WT or *K14MycER*-transgenic mice treated with 4OHT for 4 days. (J) Knockdown of *cMyc* in primary cultures of WT keratinocytes causes a decrease in *Lrig1* transcript levels. Error bars represent SD (n = 4).

Activation of Myc in the basal layer of mouse epidermis results in increased proliferation (Arnold and Watt, 2001; Waikel et al., 2001), whereas, in cultured human epidermal keratinocytes, LRIG1 overexpression decreases Myc levels and reduces proliferation (Jensen and Watt, 2006). There was upregulation of Myc protein and mRNA in the tail skin of *Lrig1*-null mice (Figure 5A). This suggests that the increased proliferation of *Lrig1*-null epidermis is attributable, at least in part, to increased Myc activity.

To examine whether Myc activation affected *Lrig1* expression, we examined the epidermis of *K14MycER*-transgenic mice following topical application of 4-hydroxy-Tamoxifen (4OHT) (Arnold and Watt, 2001). Myc activation resulted in increased *Lrig1* mRNA levels in telogen back skin (Figure 5B). There was an increase in Lrig1-expressing cells in the junctional zone, with Lrig1-positive cells extending upwards into the adjacent IFE (Figures 5D–5G).

Examination of ∼1.6 kb upstream of the *Lrig1* transcriptional start site revealed several E boxes, including a pair at 1546 and 1391 bp (Figure 5H). When the upstream sequence was coupled to luciferase and transiently transfected into *K14MycER* keratinocytes, we observed a 4OHT concentration-dependent induction of *Lrig1* promoter activity when compared to normal cells (Figure 5C).

Following chromatin immunoprecipitation (ChIP) of cells from 4OHT-treated *K14MycER* back skin, one region of the *Lrig1* promoter containing the two E boxes was specifically enriched in two independent biological replicates (Figure 5I). As a positive control, we confirmed that the promoter of *Nucleolin*, a known Myc target gene, was also enriched in cMyc binding (Figure 5I). A genomic sequence 7 kb downstream of the *Nucleolin* promoter was not bound (Figure 5I, Ctrl). In addition, knockdown of *Myc* expression in primary mouse keratinocytes using RNAi led to a comparable reduction in *Lrig1* expression (Figure 5J).

We conclude that cMyc positively regulates *Lrig1* and that the two proteins are involved in a feedback loop that controls their expression (Jensen and Watt, 2006).

### Lineage Analysis of Junctional Zone Cells

**Figure 6:**
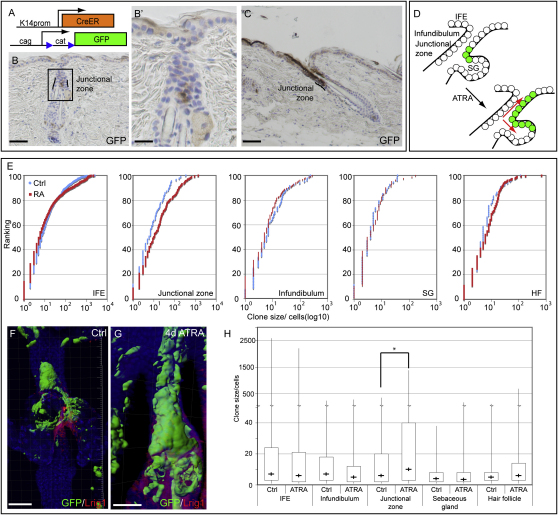
Lineage Analysis of Junctional Zone Cells (A) Schematic summary of the two mouse lines that were crossed for lineage tracing. Leakiness of Cre expression in the absence of 4OHT was exploited for clonal analysis. (B and C) Sections of back skin stained for GFP, showing two examples of junctional zone clones. (B′) Insert in (B) shown at higher magnification. (D) Schematic diagram showing contribution of junctional zone clones (green) to IFE and SG in control (left) and ATRA-treated skin. (E) Cumulative frequency plots of the size distribution of GFP-positive clones originating in the IFE, junctional zone, infundibulum, SG, and HF (below the level of the SG). Clone size (volume) was determined from 3D reconstructions of confocal images. Diagrams represent ranking based on cumulative percentage of clones as a function of clone size (exponential scale) in control (blue) and ATRA-treated (red) skin. (F and G) Three-dimensional projections of whole-mount reconstructions stained for GFP and LRIG1. Color coding indicates antibody labeling. GFP expression is shown by isosurface labeling. (F) Control. (G) Treated with ATRA. (H) Clone size distribution in control (ctrl) and ATRA-treated skin. Data from (E) are replotted as box and whisker plot of GFP clone sizes (volume) in the different epidermal regions in control and ATRA-treated tail epidermis. Minimum and maximum clone sizes are marked by whiskers; upper and lower box boundaries indicate clones in the 25th to 75th percentile, and cross (+) indicates median clone size. Asterisk indicates statistically significant change in median clone size following ATRA treatment (p < 0.05, two-tailed unpaired Mann-Whitney test). Scale bars, 100 μm (B, C, F, and G) and 25 μm (B′).

The phenotype of *Lrig1*-null epidermis (Figure 4) suggested that, although Lrig1-positive cells could contribute to all of the epidermal lineages in skin reconstitution assays (Figure 3), their role in epidermal homeostasis might be to maintain the IFE. To investigate this, we exploited the low stochastic leakiness associated with *Keratin 14* promoter-driven CreER recombination events (Hong et al., 2004; Vasioukhin et al., 1999). *K14CreER* mice were crossed with *CAG-CAT-eGFP* (*stop-flox-GFP*) mice (Kawamoto et al., 2000), in which Cre-mediated recombination results in GFP expression in the descendents of each cell in which the recombination event occurred (Bleul et al., 2006; Figure 6A). Recombination mainly occurs postnatally, and from 3 months, epidermal GFP-positive clones are readily detectable.

It has previously been reported that, under steady-state conditions, the IFE is primarily maintained by committed progenitors located within the IFE; however, any contribution of cells in the junctional zone or infundibulum was excluded from the analysis (Clayton et al., 2007). In back skin from 4-month-old *K14CreER* × *stop-flox-GFP* animals, clones of GFP-expressing cells were detected in all epidermal regions: junctional zone, infundibulum, IFE, SG, and HF. The clones of GFP cells in the junctional zone (Figure 6B) occasionally extended into the infundibulum and IFE (Figure 6C). To examine the response of the junctional zone population to increased IFE proliferation, we treated *K14CreER* × *stop-flox-GFP* mice with all-trans retinoic acid (ATRA) for 4 days, which preferentially stimulates IFE proliferation (Collins and Watt, 2008; data not shown).

Three-dimensional reconstructions of epidermal whole mounts were analyzed with Volocity software. The size (volume) of individual GFP clones located in the junctional zone (n = 85 in control [ctrl] epidermis; n = 161 in ATRA-treated epidermis), IFE (n = 1184 ctrl, 1525 ATRA), infundibulum (n = 81 ctrl, 152 ATRA), SG (n = 53 ctrl, 80 ATRA), and HF (n = 65 ctrl, 293 ATRA) was determined, and the cumulative frequency distribution of clone sizes was plotted (Figure 6E). In control epidermis, the median clone size (50% ranking in Figure 6E; “+” in Figure 6H) was similar in IFE, HF, junctional zone, and infundibulum but significantly smaller in the SG (p < 0.05, two-tailed unpaired Mann-Whitney test).

The only category of clone to enlarge significantly upon ATRA treatment was that originating in the junctional zone (Figures 6D and 6H; p < 0.05; Mann-Whitney two-tailed unpaired test). The increase was observed for all clone sizes falling between the 25th and 100th percentile (25%–100% ranking in Figure 6E). ATRA treatment also resulted in the enlargement of junctional zone clones compared with ATRA-treated clones in the IFE, infundibulum, SG, and HF (Figure 6H; p < 0.05). Both before and after ATRA treatment, junctional zone clones contributed cells to the infundibulum, IFE, and SG, but not to the HF below the SG (Figures 6F and 6G). Conversely, HF clones never contributed to the junctional zone.

These results indicate that, although Lrig1-positive cells give rise to HF lineages in skin reconstitution assays (Figure 3), they are bipotent in intact skin (Figure 6D). In response to an IFE proliferative stimulus, there is selective expansion of junctional zone clones into the IFE and SG (Figure 6D).

### Loss of *Lrig1* Results in Expansion of the Junctional Zone Stem Cell Compartment and Increased β-Catenin Responsiveness

**Figure 7:**
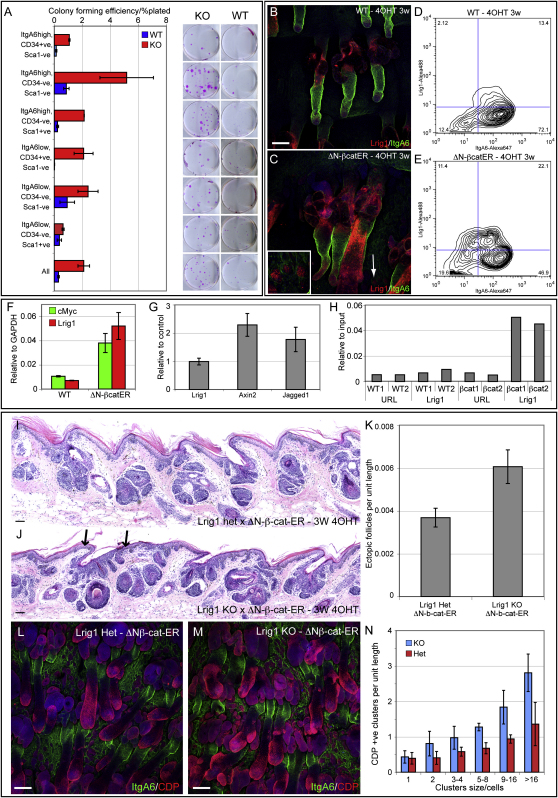
Effect of *Lrig1* Loss on Stem Cell Renewal and Responsiveness to β-Catenin Activation (A) Clonal growth assays of primary keratinocytes sorted based on CD34, Sca1, and α6 integrin (Figure 3A–C) from the skin of wild-type (WT) and *Lrig1*-null (KO) adult littermates. Error bars represent SD (n = 3). Six-hundred Sca1-negative cells and 2500 Sca1-positive and unfractionated cells were seeded; representative dishes are shown. (B and C) Tail epidermal whole mounts of WT and *K14ΔNβ-cateninER* mice treated with 4OHT for 3 weeks. Arrow and insert indicate Lrig1-positive ectopic follicles in interfollicular epidermis. (D and E) Flow cytometric analysis for Lrig1 and α6 integrin of cells from WT and *K14ΔNβ-cateninER* mice treated with 4OHT for 3 weeks. The proportion of cells in each fraction is indicated. (F) Q-PCR of *Lrig1* and *cMyc* mRNA in back skin of WT and *K14ΔNβ-cateninER*-transgenic mice treated with 4OHT for 2 weeks. Expression levels are relative to *Gapdh*, and error bars represent SD (n = 3). (G) Q-PCR of levels of *Lrig1*, Axin2, and Jagged1 in primary murine epidermal keratinocytes treated with Wnt3A. Data are expressed relative to unstimulated cells. Error bars represent SD (n = 3). (H) ChIP analysis of endogenous cMyc on the *Lrig1* promoter in WT and *K14ΔNβ-cateninER* mice treated with 4OHT for 10 days. Data represent two separate samples and show level of isolated genomic DNA relative to amount of input DNA. (I–M) Hematoxylin and eosin stained sections (I and J) and whole mounts (L and M) of adult tail epidermis from *K14ΔNβ-cateninER* × *Lrig1* heterozygous (het) or knockout (KO) mice treated with 4OHT for 3 weeks. The number of ectopic HFs formed from the interfollicular epidermis was scored by morphology (K) and by the appearance of clusters of CDP-expressing cells (N) (two independent experiments; KO, n = 8; WT, n = 7). Arrows in (J) indicate expanded infundibulum with associated ectopic follicles. In (B), (C), (L), and (M), color coding indicates antibody labeling. Scale bars, 100 μm (B and C) and 200 μm (I, J, L, and M).

To examine the effects of *Lrig1* loss on keratinocyte growth in culture, we performed clonal growth assays on the six epidermal populations sorted on the basis of CD34, Sca1, and α6 expression from the back skin of telogen mice (Figure 7A). There was an increase in the colony-forming efficiency of each population and also of total basal cells (all: α6 positive, low forward, and side scatter). This is consistent with expression of *Lrig1* in all subpopulations of cells, albeit at different levels (Figure S2E), and with upregulation of cMyc in cultured keratinocytes (Figure 5 and data not shown). However, the increase was most dramatic in the CD34-negative, Sca1-negative, α6 integrin-high population, the cells that normally are highly enriched for *Lrig1* expression. We conclude that, in the absence of Lrig1, this population of cells has a selective growth advantage.

The expansion of clonal growth on loss of *Lrig1* is reminiscent of the effects of activating β-catenin (Silva-Vargas et al., 2005). Therefore, we examined Lrig1 expression in *K14ΔNβ-cateninER*-transgenic mice, in which β-catenin is activated by 4OHT treatment (Lo Celso et al., 2004; Silva-Vargas et al., 2005). β-catenin activation resulted in an increase in Lrig1-positive cells in the junctional zone and adjacent IFE (Figures 7B and 7C), as observed in *K14MycER* mice (Figures 5F and 5G). Lrig1 was also expressed in ectopic follicles (Figures 7C, arrow and insert). There was a 2-fold increase in the α6 integrin-high, Lrig1-positive population (Figures 7D and 7E) and a 4-fold increase in the CD34-negative, Sca1-negative, α6-high population (population 2 in Figures S2G and S2H).

Q-PCR analysis showed an increase in *Lrig1* mRNA in response to β-catenin (Figure 7F). However, 5 kb upstream of the *Lrig1* transcription start site did not contain TCF- and Lef-binding sites, and Lrig1 expression was unaffected when β-catenin signaling was blocked with an N terminally truncated Lef1 transgene (data not shown). *Lrig1* expression was not induced in keratinocytes by Wnt3A under conditions in which known target genes, *Axin2* and *Jagged1* (Estrach et al., 2006; Lustig et al., 2002), were induced (Figure 7G). In contrast, *cMyc*, a known target of β-catenin (He et al., 1998) was upregulated upon activation of β-catenin (Figure 7F). Moreover, upon β-catenin activation, there was a substantial increase in the amount of endogenous Myc associated with the E boxes on the *Lrig1* promoter (Figure 7H). We conclude that, upon β-catenin activation, the Lrig1-positive compartment expands, but this reflects upregulation of cMyc because *Lrig1* expression is not directly regulated by β-catenin.

To examine the consequences of loss of *Lrig1* on β-catenin activation, we crossed *K14ΔNβ-cateninER* mice with *Lrig1*-null mice and examined adult tail epidermis following 4OHT treatment. On a wild-type background, 4OHT treatment induces ectopic HFs in the IFE, SGs, and existing follicles (Silva-Vargas et al., 2005). Activation of β-catenin in the absence of *Lrig1* caused expansion of the junctional zone and a selective increase in ectopic follicles in the IFE, particularly in the vicinity of the junctional zone (Figures 7I and 7J). *Lrig1* loss led to some SG enlargement on β-catenin activation, but there was no increase in the number of ectopic follicles arising from the SG or existing follicles (Figures 7I, 7J, 7L, and 7M).

In addition to quantitating the ectopic follicles per unit length of IFE (Figure 7K), we scored epithelial cell clusters expressing CDP, an early marker of ectopic follicle formation (Silva-Vargas et al., 2005) (Figure 7N). There was an increase in CDP-positive clusters, irrespective of cluster size (Figure 7N). Thus, *Lrig1* loss affects ectopic follicle number rather than size.

We conclude that activation of β-catenin in the absence of *Lrig1* causes an increase in the number of junctional zone stem cells and that they can form ectopic HF.

## Discussion

We have defined a distinct population of multipotent stem cells in the junctional zone of the HF that expresses Lrig1. In epidermal reconstitution experiments, these cells contribute as effectively as bulge stem cells to all of the epidermal lineages. However, lineage analysis suggests that they are bipotent in steady-state epidermis, giving rise to cells in the IFE and SG.

It has been reported that, during normal epidermal homeostasis, the IFE can be maintained by committed progenitors (Clayton et al., 2007). Following wounding, bulge stem cells contribute only transiently to the IFE (Ito et al., 2005), whereas cells originating above the bulge stem cell compartment or in the infundibulum contribute long-term (Levy et al., 2007). Several lines of evidence suggest that the junctional zone constitutes this previously unidentified reservoir of IFE stem cells. Lineage tracing demonstrates that cells in the junctional zone contribute to the infundibulum, SG, and, occasionally, the IFE. In response to ATRA, which causes IFE proliferation, there is selective expansion of junctional zone clones into these compartments. Moreover, activation of β-catenin causes a marked increase in the size of the junctional zone compartment. In *Lrig1*-null epidermis, this expansion is associated with a corresponding increase in the number of ectopic HFs that form specifically in the IFE adjacent to the infundibulum, but not elsewhere. This cannot simply be due to increased IFE proliferation in *Lrig1*-null epidermis because ATRA treatment, which stimulates IFE proliferation, does not affect β-catenin-induced ectopic HF formation (Collins and Watt, 2008; C.A.C. and F.M.W., unpublished data).

The Lrig1-positive population is specified early in development, coinciding with the formation of the HF stem cell niche (Nowak et al., 2008), and persists during adulthood. Lrig1-expressing stem cells can be isolated directly via Lrig1 or enriched on the basis of high α6 integrin expression and lack of CD34 and Sca1. Cells that express Lrig1 are negative for the bulge markers keratin 6a, keratin 15, CD34, and Lgr5 (Liu et al., 2003; Trempus et al., 2003; Jaks et al., 2008). They show elevated expression of *Blimp1*, a previously reported marker of SG stem cells (Horsley et al., 2006), and of the *Lgr* family member *Lgr6* (Hsu et al., 2000). Expression of *MTS24/Plet1*, which is reported to be a marker of Sca1-negative, CD34-negative keratinocytes (Jensen et al., 2008; Nijhof et al., 2006), was not specifically upregulated in Lrig1-expressing cells. Lrig1 is the first marker of the junctional zone to be described, and cells in this region have a unique gene expression profile.

Lrig1 was originally identified as a human IFE stem cell marker that keeps cells in a quiescent, nondividing state (Jensen and Watt, 2006). Consistent with those observations, loss of *Lrig1* in mouse epidermis resulted in hyperproliferation in the IFE. *Lrig1* loss did not affect the HFs or SGs. In contrast, loss of the transcriptional repressor *Blimp1* selectively induces excessive proliferation in the SG (Horsley et al., 2006), whereas loss of *NFATc1* causes continuous HF cycling (Horsley et al., 2008). Loss of *Lrig1*, *Blimp1*, or *NFATc1* is characterized by reduction in the number of LRCs as they are recruited into the cell cycle. Thus, it appears that these three proteins function in concert to maintain quiescence in different epidermal stem cell compartments.

We found that Lrig1 and Myc participate in an autoregulatory feedback loop; cMyc regulates transcription of the *Lrig1* gene, and Lrig1 negatively regulates Myc expression (Figure 6; Jensen and Watt, 2006). It has also been shown that Blimp1 is a direct negative regulator of Myc (Horsley et al., 2006). This may explain why ectopic activation of Myc under the control of the keratin 14 promoter causes hyperplasia of both IFE and SG (Arnold and Watt, 2001; Waikel et al., 2001), whereas loss of *Lrig1* specifically affects the IFE. It is interesting that Myc is required for anagen entry (Soucek et al., 2008) because this coincides with the appearance of the ring of Lrig1-positive cells above the HF bulb (Figure 1G).

In epidermal reconstitution experiments, Lrig1-enriched cells and Lrig1-positive cells were as effective as bulge cells in contributing to the different epidermal lineages. Surprisingly, Sca1-positive cells, previously reported to be committed progenitors of the IFE (Jensen et al., 2008), were also capable of contributing to HFs and SGs. We suggest that the behavior of cells in different regions of the epidermis is normally constrained by signals from their local environment (Owens and Watt, 2003). However, most, if not all, epidermal cells that are competent to proliferate will exhibit multilineage differentiation potential when challenged genetically (Silva-Vargas et al., 2005), by wounding (Ito et al., 2007), or in epidermal reconstitution experiments. This would certainly be consistent with recent observations on the properties of cells from different regions of the ocular surface (Majo et al., 2008). Important questions for the future are whether all of the different epidermal stem cell populations arise from the P-cadherin-low, Lrig1-positive cells in developing HFs and whether the different stem cell populations in adult epidermis have a hierarchical relationship or are completely autonomous.

## Experimental Procedures

### Mice and Labeling Experiments

*Lrig1*-null (Suzuki et al., 2002), *K14MycER* (Arnold and Watt, 2001), *K14ΔN-β-cateninER* (line D2) (Lo Celso et al., 2004), *K14CreER* (Hong et al., 2004; kind gift from B. Stripp), *CAG-CAT-eGFP* (Kawamoto et al., 2000; kind gift from J. Miyazaki), and *β-actin-eGFP* (C57BL/6 jax strain, stock number: 003291) mice have been described previously. In some experiments, mice were treated with 4-hydroxy-Tamoxifen (4OHT) at a concentration of 1.5 mg/200 μl in acetone three times a week on the back and tail. Some mice were treated with 100 μl 0.5mM ATRA (Sigma) in acetone on back and tail as described (Collins and Watt, 2008). DNA label-retaining cells were generated by repeated BrdU injections of neonatal mice, as described previously (Braun et al., 2003).

### Flow Cytometry

Keratinocytes were isolated from telogen dorsal back skin using either trypsin as described (Silva-Vargas et al., 2005) or thermolysin (Walzer et al., 1989). To isolate epidermal cells using thermolysin, we rinsed back skin in 10% Betadine and 70% ethanol and washed it in PBS. The dermal side was thoroughly scraped to remove excess fat, and then the tissue was floated in 0.25 mg/ml Thermolysin (Sigma) in calcium-free FAD medium for 1 hr at 37°C. The epidermis was subsequently scraped from the dermis, minced with scalpels, and disaggregated by gentle pipetting. Thermolysin was inactivated by addition of FCS, and the cells were pelleted and resuspended for labeling with antibodies (see Supplemental Data). Cell sorting was carried out using a MoFlo high-speed sorter (Dako Cytomation), and analysis was carried out on a CyAN ADP analyzer (Dako Cytomation). All data analysis was conducted using the FlowJo program.

### Hair Reconstitution Assays

The experiments were performed essentially as described previously (Jensen et al., 2008). Each graft consisted of 10^5^ GFP-expressing epidermal cells mixed with 3 × 10^6^ unlabeled, unsorted epidermal cells and 5 × 10^6^ dermal cells isolated from neonatal fibroblasts. We typically isolated 10^5^ cells per mouse of Lrig1-positive cells or the two groups of α6 integrin-high, Sca1-negative cells. The tops of the graft chambers were removed 1 week after grafting, and the chambers were removed 1 week later. Hair growth was typically observed 1 week after chamber removal. Mice were sacrificed and analyzed 4–5 weeks postgrafting.

### Mouse Keratinocyte Culture

Mouse keratinocytes were isolated and cultured on a J2 3T3 feeder layer as described (Silva-Vargas et al., 2005). For clonal growth assays, 600 to 2500 cells were plated per well in 6-well plates and cultured for 2 weeks prior to fixation and staining with 1% Rhodamine B.

### Statistical Analysis

The significance of quantitative data was tested using the unpaired, two-tailed Student's t test. Data from the lineage analysis experiments were analyzed using an unpaired, two-tailed Mann-Whitney test.

## Supplemental Data

Document S1. Supplemental Experimental Procedures and Two Figures
