# Supplementary material for: Lrig1 Expression Defines a Distinct Multipotent Stem Cell Population in Mammalian Epidermis
Source: Cell Stem Cell. 2009 May 8;4(5):427–39. doi: 10.1016/j.stem.2009.04.014 (PMC2698066; doi:10.1016/j.stem.2009.04.014)
Supplement: Document S1. Supplemental Experimental Procedures and Two Figures [file mmc1.pdf]

Cell Stem Cell, ***Volume 4***

Supplemental Data

Lrig1 Expression Defines a Distinct Multipotent Stem Cell Population in Mammalian Epidermis

Kim B. Jensen, Charlotte A. Collins, Elisabete Nascimento, David W. Tan, Michaela Frye, Satoshi Itami, and Fiona M. Watt

Supplemental Experimental Procedures

Flow Cytometry

Cells were labelled with the following fluorochrome-conjugated antibodies: $\alpha 6$ integrin (FITC, PE or Alexa647, GoH3, Serotec, Germany), Sca1 (PE or Alexa700, Clone D7, eBiosciences), CD34 (Biotin, Pacific Blue, Alexa647, RAM34, eBiosciences) and Lrig1 (R & D Systems). Cells were incubated with a cocktail of the antibodies for 45 minutes at 4^0C before washing twice in PBS. Certain combinations of antibodies were visualised with streptavidin PE (Biotin CD34) or, for detection of Lrig1, anti-goat Alexa488 or 647 (Invitrogen). Cell viability was assessed by 7AAD labelling (BD Biosciences).

Cells were sorted with a MoFlo high-speed sorter (Dako Cytomation) on the basis of Lrig1, $\alpha 6$ integrin, CD34 and Sca1 labelling (Jensen et al., 2008), after gating out dead cells and differentiated cells (high forward and side scatter; Jones and Watt, 1993). For grafting experiments GFP positive cells were sorted following labelling with $\alpha 6$ integrin-PE, Sca1-Alexa 700 and CD34-Alexa647 conjugated or with Lrig1 anti-goat Alexa647 antibodies.

Flow analysis of BrdU labelled cells was performed with the APC-BrdU flow cytometry kit (BD biosciences) in combination with CD34-pacific blue, $\alpha6$ integrin-FITC and Sca1-PE conjugated antibodies.

Antibodies and Immunolabeling

The source of antibodies used has been described previously (Braun et al., 2003; Palmer et al., 2008; Silva-Vargas et al., 2005), except for the antibodies to Lrig1 (goat polyclonal, R and D), P-cadherin (PCD1, Invitrogen), Sca1 (eBiosciences), GFP (chicken polyclonal, Abcam) and CDP (Santa Cruz). Conventional frozen and paraffin sections as well as tail epidermal whole mounts were prepared and immunolabelled as described previously (Braun et al., 2003; Lo Celso et al., 2004).

Tissue grafts were imaged using a Leica M165 FC stereo microscope. Confocal images were acquired on a Leica TCS SP5 confocal microscope. Z-stacks were acquired at 200Hz with an optimal stack distance and 1024x1024 dpi resolution. Z-stack projections were generated using the LAS AF software package (Leica Microsystems).

Image Analysis

Confocal 3D images were imported into Volocity 5.0 high performance 3D imaging software (Perkin Elmer) for accurate measurement of GFP clone volumes in 3-dimensions. Clones were initially identified by thresholding the maximum intensity of the GFP channel, and restricting measurements to areas greater than $80\mu m^3$ (the approximate volume of a single cell) to

eliminate background fluorescence. Clones were subsequently categorised according to whether they lay completely in the IFE, whether they lay in the infundibulum, with or without additional IFE contribution (infundibulum clone); contained junctional zone cells, with or without additional contribution to the IFE, SG or infundibulum (junctional zone clone); lay exclusively in the SG (SG clone); or resided in the HF beneath the junctional zone. Four representative wholemount images acquired at 20x magnification were analysed for each of three animals in control and ATRA treated groups. Images of isosurfaced GFP clones were generated using Imaris 6.2 (Bitplane).

RNA Isolation and Quantitative Real-Time PCR

RNA was isolated from whole skin using TriZol (Collins and Watt, 2008), and from primary cells using SV total RNA isolation kit (Promega). RNA from whole skin was treated with RQ1 RNase free DNase (Promega) before reverse transcription using Superscript III (Invitrogen) and random hexamer primers. Specific gene expression assays from Applied Biosystems were used for amplification using an Applied Biosystems 7900HT Real Time PCR System (Applied Biosystems, Foster USA) (Probe IDs available on request). Samples were normalised to GAPDH using the ΔCt method.

Chromatin Immunoprecipitation

Primary mouse keratinocytes were isolated from dorsal skin of K14MycER mice and littermate controls treated with 4OHT for 4 days or K14DN-β-cateninER mice and littermate controls treated with 4OHT for 10 days. Samples were pooled from at least 6 mice for 1 ChIP experiment. ChIP

experiments for the MycER samples were performed as described (Odom et al., 2007), and for the K14ΔNβ-cateninER samples according to the Manufacturer's instructions (Upstate) with a c-Myc antibody (N262, Santa Cruz). Quantitative Real Time PCR was performed using SYBR Green and primers were designed to amplify the Lrig1 promoter region containing the 2 E-boxes (Lrig1-forward: 5'-GAC AGC CAC GTT TAG CAT CA-3'; Lrig1-reverse: 5'-GGG AAG AAA AAC ACC GAA CA-3'), the nucleolin promoter (nucleolin-forward: 5'-GGC TGG AAG CGA GAG AAA G-3'; nucleolin-reverse: 5'- TCA CCT CTT AAA GCA GCA CCA-3') and a region located 7000bp upstream of the nucleolin promoter (negative control-forward: 5'- GCTGGCC TCAAACTCAGAAA-3'; negative control-reverse: 5'-GGCGCACACCTT TAATCC-3'). Samples were amplified as above and quantified with a calibration line performed with control DNA

Manipulation of Mouse Keratinocyte Cultures

For Myc knock down experiments, primary mouse keratinocytes were cultured to 50% confluence. The feeders were removed and the keratinocytes were incubated overnight in supplemented KSFM (Invitrogen). The following day cells were incubated with 40pmol of smart pool RNAi (Dharmacon for Myc RNAi and Qiagen for scrambled RNAi) in Lipofectamine 2000 (Invitrogen) for 3 hours. Cells were cultured for 48 hours in KSFM and then RNA was isolated using SV total RNA isolation kit (Promega).

To activate β-catenin signalling, primary mouse keratinocytes (P1-P4) were cultured to 80% confluence and starved overnight in 0.5% FBS supplemented

FAD medium. Cells were treated with purified Wnt3A as described previously (Pálmer et al., 2008) for 6 hour in calcium-free FAD medium containing 0.5% FBS, then lysed. RNA was purified using an SV total RNA isolation kit (Promega).

Western Blotting

Tail skin was snap frozen, transferred to 1 x TBS supplemented with 2 x proteinase and phosphatase inhibitor cocktails (Roche) and homogenised for 30 seconds in a Polytron PT-MR2100 (Kinematic AG, Switzerland). 2 x RIPA buffer was subsequently added and samples were incubated on ice for 20 minutes. Equal amounts of protein from Lrig1 heterozygous and null littermates were analysed by Western blotting using antibodies to cMyc (N262, Santa Cruz) and β-tubulin (Sigma).

Promoter Analysis

The Lrig1 promoter was cloned from 1577bp upstream of exon1 to the start codon and inserted into the XhoI and NcoI sites of pGL3 basic. Keratinocytes from wild type and K14MycER transgenic mice were transiently transfected with the promoter construct as well as pRL–TK renilla. After overnight recovery cells were treated with 0-50 nM 4OHT for 24 hours in calcium-free FAD in the presence of 0.5% fetal calf serum. Luciferase activity was measured using a dual luciferase reporter assay (Promega) on a Glomax (Promega).

Supplemental References

Braun, K. M., Niemann, C., Jensen, U. B., Sundberg, J. P., Silva-Vargas, V., and Watt, F. M. (2003). Manipulation of stem cell proliferation and lineage commitment: visualisation of label-retaining cells in wholemounts of mouse epidermis. Development 130, 5241-5255.

Collins, C. A., and Watt, F. M. (2008). Dynamic regulation of retinoic acid-binding proteins in developing, adult and neoplastic skin reveals roles for beta-catenin and Notch signalling. Dev Biol 324, 55-67.

Jensen, U. B., Yan, X., Triel, C., Woo, S. H., Christensen, R., and Owens, D. M. (2008). A distinct population of clonogenic and multipotent murine follicular keratinocytes residing in the upper isthmus. J Cell Sci 121, 609-617.

Jones, P. H. and F. M. Watt (1993). Separation of human epidermal stem cells from transit amplifying cells on the basis of differences in integrin function and expression. Cell 73: 713-24.

Lo Celso, C., Prowse, D. M., and Watt, F. M. (2004). Transient activation of beta-catenin signalling in adult mouse epidermis is sufficient to induce new hair follicles but continuous activation is required to maintain hair follicle tumours. Development 131, 1787-1799.

Odom, D. T., Dowell, R. D., Jacobsen, E. S., Gordon, W., Danford, T. W., MacIsaac, K. D., Rolfe, P. A., Conboy, C. M., Gifford, D. K., and Fraenkel, E. (2007). Tissue-specific transcriptional regulation has diverged significantly between human and mouse. Nat Genet 39, 730-732.

Palmer, H. G., Anjos-Afonso, F., Carmeliet, G., Takeda, H., and Watt, F. M. (2008). The vitamin D receptor is a Wnt effector that controls hair follicle

differentiation and specifies tumor type in adult epidermis. PLoS ONE 3, e1483.

Silva-Vargas, V., Lo Celso, C., Giangreco, A., Ofstad, T., Prowse, D. M., Braun, K. M., and Watt, F. M. (2005). Beta-catenin and Hedgehog signal strength can specify number and location of hair follicles in adult epidermis without recruitment of bulge stem cells. Dev Cell 9, 121-131.

Supplemental Figures

Figure S1

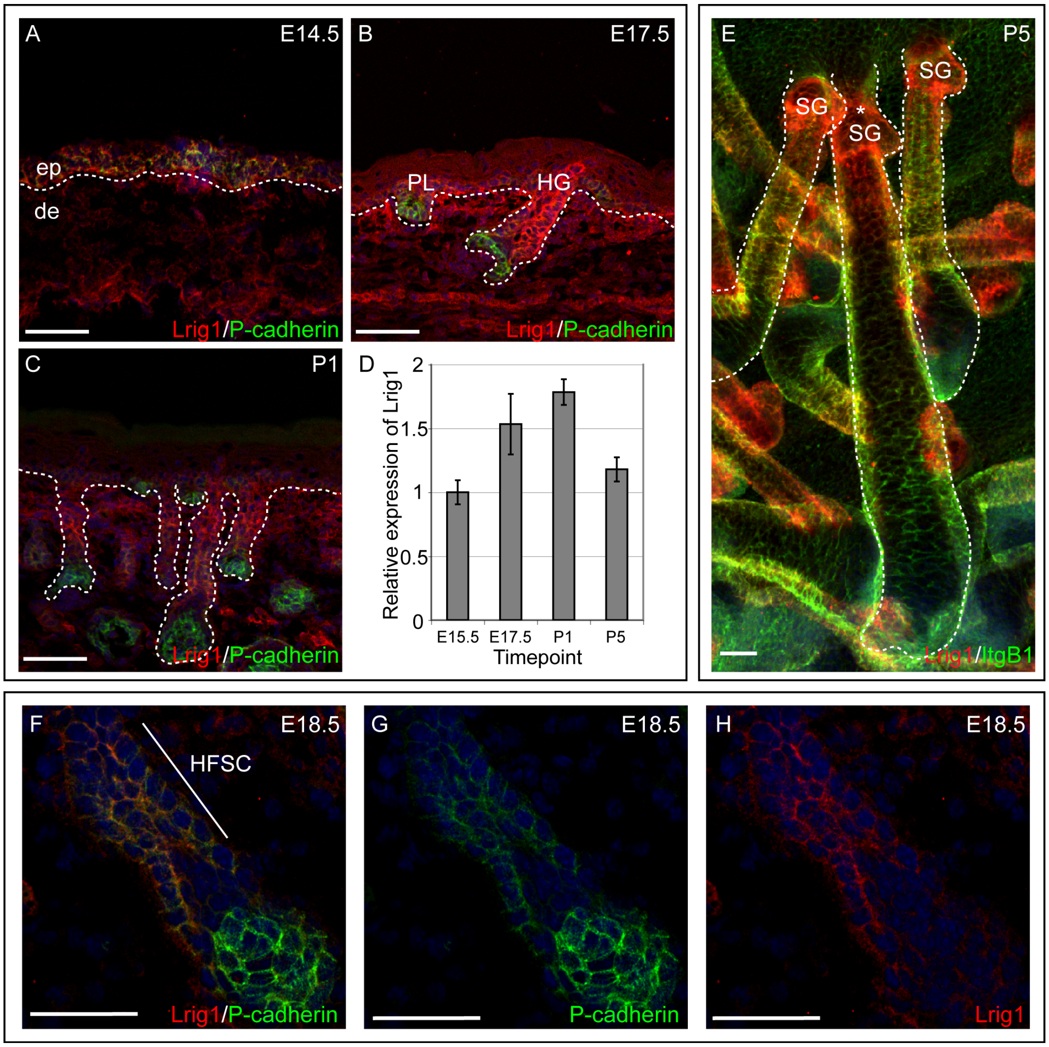

Figure S1. Lrig1 Expression during Embryonic Development

(A-C, F-H) Sections of embryonic (A: E14.5; B:E17.5; F-H: E18.5) and postnatal (C: P1) skin and (E) tail epidermal whole mount (P5). Colour coding indicates antibody labelling. Dashed lines represent boundary between dermis and epidermis (A-C) or demarcate hair follicles and sebaceous glands (E). ep: epidermis; de: dermis; PL: hair follicle placode; HG: hair germ, SG: sebaceous gland. Scale bars: 25µm (A-C), 50µm (E-H). (D) Q-PCR of Lrig1 mRNA levels in back skin at different developmental stages. Lrig1 was

normalised to GAPDH and then to expression at E15.5 (n=3). (F) is merge of (G, H), showing Lrig1 that P-cadherin dim prospective hair follicle stem cells (HFSC) above the bulb express Lrig1. (F-H) are high magnification views of Figure 1A.

Figure S2

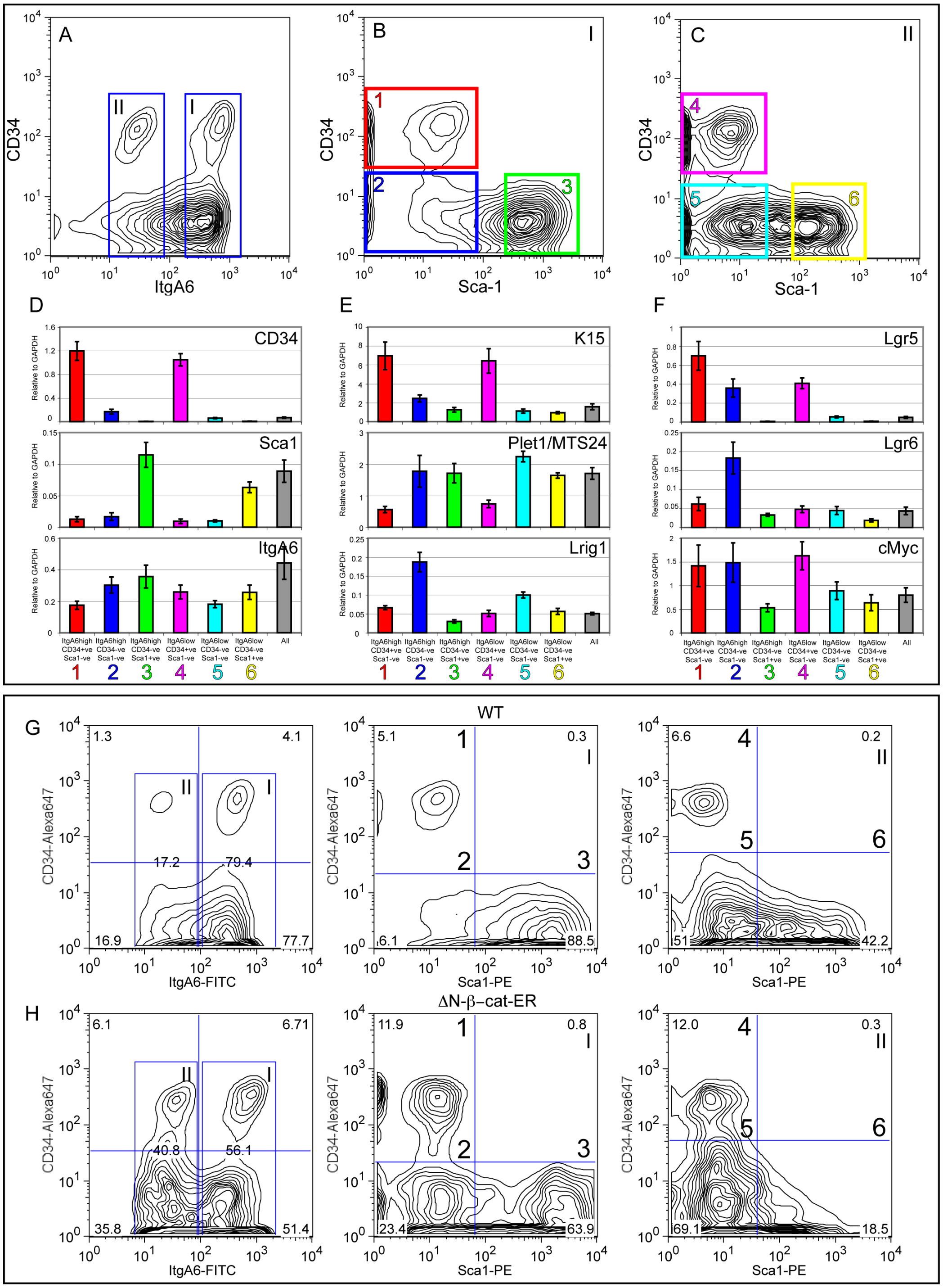
A
B
C
D
E
F
G
H
CD34
ItgA6
Sca-1
CD34
K15
Lgr5
Sca1
Plet1/MTS24
Lgr6
ItgA6
Lrig1
cMyc
Relative to GAPDH
ItgA6high CD34+ve Sca1-ve
ItgA6high CD34-ve Sca1-ve
ItgA6high CD34-ve Sca1+ve
ItgA6low CD34+ve Sca1-ve
ItgA6low CD34-ve Sca1-ve
ItgA6low CD34-ve Sca1+ve
All
WT
ΔN-β-cat-ER
CD34-Alexa647
ItgA6-FITC
Sca1-PE

Figure S2. Isolation and RNA Profiling of Lrig1-Expressing Cells Selected on the Basis of CD34, α6 Integrin and Sca1 Levels

(A-C) and the CD34 and Lrig1 profiles in (D, E) are also shown in Figure 3A-D. (A-C) Keratinocytes isolated from the back skin of wild type adult telogen (7 week old) mice were labelled with Sca-1-PE, α6 integrin–FITC and CD34-Alexa647 and subjected to high speed flow sorting. Dead cells (7AAD positive) and differentiated cells (high forward and side scatter) were gated out and cells were sorted into α6 integrin high (I) and low (II) populations (A). Populations I (B) and II (C) were gated into three further populations on the basis of Sca-1 and CD34 expression, yielding a total of 6 discrete populations of keratinocytes (1-3 in B; 4-6 in C). (D-F) RNA from 10^5 cells of each of the 6 populations or 10^5 total live cells with low forward/side scatter (all) were analysed using quantitative PCR for the genes indicated. Data are means ± SEM from cells of 5 mice. (G, H) Fractionation of adult epidermal cells into the six populations shown in (A-C) from WT (G) or K14ΔNβ-cateninER mice (H). Mice were treated with 4OHT for 3 weeks prior to harvesting. Proportion of cells in each fraction is indicated.
